# Health related quality of life of immigrant children: towards a new pattern in Germany?

**DOI:** 10.1186/1471-2458-14-790

**Published:** 2014-08-03

**Authors:** Ester Villalonga-Olives, Nicole von Steinbüchel, Claudia Witte, Erich Kasten, Ichiro Kawachi, Christiane Kiese-Himmel

**Affiliations:** Department of Medical Psychology and Medical Sociology, University Medical Center, Georg-August-University, Göttingen, Germany; Department of Social and Behavioral Sciences, Harvard School of Public Health, Boston, MA 02215 USA

**Keywords:** Health related quality of life, Kindergarten children, Life course, Migrants, Migrant health paradox

## Abstract

**Background:**

To study Health related quality of life (HRQoL) of a sample of kindergarten children with migration background.

**Methods:**

Five kindergartens in Frankfurt/Main and Darmstadt (Germany) participated. HRQoL was measured with the Kiddy-KINDL (KK) in 3 to 5 year old children. We examined the associations of HRQoL with socio-demographic variables, positive development and resilience, socio-emotional and motor development. Linear regression models were applied to examine differences in HRQoL between migrant and native-born German children.

**Results:**

The response rate was 90.5% (N = 283). The children had predominantly migrant background (81.35%). Perceived health was slightly higher in migrants (69.85, SD 17.00) compared to native-born German children (68.33, SD 17.31, p > 0.05), even though migrant children were characterized by a lower socio-economic status (p < 0.01).

**Conclusions:**

Results suggest that HRQoL at early ages in our study exhibits a different pattern than reported previously in studies among older individuals. We attribute the discrepancy partly to a possible changing pattern of migration in Europe with more migrants capable to migrate with healthy profiles, and to the age of our population. Our findings underscore the need to study the life course trajectory of HRQoL among young immigrants and replication in representative samples.

## Background

Migration has increased since the beginning of the worldwide economic crisis in 2008, and it is a topic of bigger interest [1]. Within the European Union (EU), data suggest that children with migrant backgrounds have lower health status than native-born populations [2–5]. These differences have been attributed to the lower socio-economic backgrounds of economic migrants, lack of social integration, deficits in second language competence and performance as well as to experiences of discrimination [6]. This pattern is in contrast to studies conducted in the United States where immigrants – particularly Mexican-Americans – have been found to have better health outcomes (e.g. a lower number of children with low birth weight rates) compared to the native-born, in spite of their lower socio-economic backgrounds.

The so-called “Latino health paradox” in the United States has been in turn ascribed to selective migration [7–10]. In other words, economic migrants constitute a healthy group of individuals who are fit to work, and often exhibit better health status than native-born individuals in spite of their socio-economic disadvantage. However, more recent migrants into the US tend to be families with job skills [11]. Considering the evolving reasons for migration over time (the “migration regime”), we hypothesize that the profile of migrants may have changed, making the patterns more similar to that observed in the United States with a profile of people with low socio-economic factors but with good job skills. Facts like the south-north migration, the levels of unemployment in the EU, and the good health status reported by migrants from Eastern and Mediterranean countries in some studies can be related to this phenomenon [12].

In contrast to the force of selective migration, there are opposite tendencies that tend to lower the health of migrant groups. Thus the migration process itself has been linked to family stress and lack of social support in the offspring of migrant families [13, 14]. But the effect of the migration process can also vary depending on the age of the children. Health related quality of life (HRQoL) is a concept which represents a person’s own perception of his or her subjective health status, functioning, and well-being in the domains of physical, psychological, social and role performance [15, 16]. Given the rise in immigrant populations, and the suggestion of a possible recent change in the migration pattern across the EU, it is of interest to measure the impact of the migratory experience on health and HRQoL of migrants, especially children.

Germany is one of the countries that received an increasing number of migrants from other countries seeking work. There are 1.14 million children under the age of five from migrant backgrounds currently living in Germany [17], representing 34.84% of children in that country. Studies have found that there are HRQoL differences between migrants and native-born depending on age, gender and socio-economic status [18–20]. Schenk and co-authors noted in 2008 that migrant parents in Germany rated the HRQoL of their children (aged 3-10 years) significantly lower than native-born parents [21]. However, there are only very few studies in European countries that compare HRQoL in children of migrant versus native-born populations [20, 22]. Indeed, there are neither reports with recent data; nor data from young children using self-reports.

In Germany, 34.84% of children between 3 to 5 years old have migrant backgrounds. In metropolitan areas, the prevalence is even higher and ranges from 45 to 65% [23]. However, there are no studies yet that have investigated the HRQoL of this population; our objective was therefore to study a sample of migrant children. We hypothesized that young migrant children would report lower HRQoL than native-born children because of the stress of dislocation as well as their disadvantaged social background. We hypothesized that HRQoL is influenced by socio-economic variables, positive development and resilience, socio-emotional and motor development, and that these will differ between migrants and native-born.

## Methods

### Design, sample and procedure

The population of the study comprised the baseline data of a longitudinal study whose main aim was to investigate health outcomes in children from 3 to 5 years old with predominantly migrant backgrounds, and to assess the development of these children. The baseline study was conducted in Frankfurt/Main and Darmstadt, Germany. Five kindergartens located in five areas of education pertaining to different neighborhoods accepted to take part in the study. Participants were enrolled at the kindergartens and 96% of the parents consented to take part in the study (N = 300). Among these children, 81.35% had at least one parent coming from another country (second generation migrants). Data collection started in May 2009 and it is still ongoing. The response rate out of the whole was 90.5% (N = 283). The project was approved by the local ethics committee of the University Medical Center, Göttingen, Germany.

Informed consent was obtained from participating families, with the approval of the kindergarten councils. Families received detailed information regarding the background and implementation of the study and were offered the opportunity to withdraw the participation of their children at any time. Interviewers were trained in person by the scientists who designed the study project and had a manual to follow the detailed instructions. The collection of data was performed using face to face interviews with children, parents and teachers, separately.

### Measures

#### Socio-demographic and socio-economic variables

We collected information about occupation and level of education of the main sustainer of the family to characterize the family’s socio-economic status. Here the international ISCO categorization was followed. To perform the analysis of these data, we created a final categorical variable: out of work (0), unskilled workers (1), skilled workers (2), professionals (3) and professionals with advanced qualifications (4). Socio-economic status data were collected by psychologists and educators during an interview with the parents.

#### Kiddy-KINDL (KK)

The Kiddy-KINDL is a validated and reliable instrument developed in Germany to measure HRQoL in children aged between 4 and 7 years [24–26]. The recall period of the questionnaire is the past week. The short version of this questionnaire includes 12 items belonging to 6 dimensions: physical well-being, psychological well-being, self-esteem, family, friends, and everyday functioning at the kindergarten. Response categories are arranged on a 3-point Likert scale (never; sometimes; very often). Scale scores of the KK range from 0 to 100, with higher scores indicating better HRQoL. We administered the self-report version of the KK to children aged 3 to 5 years. Psychometric properties of the instrument were found to be acceptable in this sample [Villalonga-Olives E, Witte C, Almansa J, Lange B, Hacker K, Dusilova I, Kiese-Himmel C, Von Steinbüchel N: Self-reported health-related quality of life in kindergarten children: psychometric properties of the Kiddy-KINDL. Submitted]. The KK was administered by psychologists and educators using face-to-face interviews with the children. The interviewers were previously trained.

#### Positive development and resilience in daily life of kindergarten children (PERIK)

The PERIK is a German instrument designed to observe and assess the preschool children’s well-being in early childhood settings of children aged 3.5 to 5 years of age. The questionnaire contains 36 items, distributed in 6 scales of 6 items each: interpersonal skills, self-monitoring/thoughtfulness, assertiveness, resilience, task oriented behavior, and enjoyment in exploratory behavior. The responses are arranged along a five-point rating-scale (always; mostly; sometimes; rarely; never). The values range from 0 to 30. Higher scores indicate better performance. The instrument has been demonstrated to have acceptable psychometric properties [27]. We have used the scale of PERIK that measures positive development and resilience, because it has been related to the concept of HRQoL and the experience of major life events like migration; the KK does not contain this facet. PERIK was answered by the kindergarten teachers.

#### Vienna developmental test (WET)

The WET is a widely used instrument which measures the developmental status of children aged 3 to 6 years [28]. The WET consists of 13 subtests and a parent questionnaire, covering 6 functional areas of development (visual, motor, learning/memory, cognitive stage, language, and socio-emotional developmen). The variables range from 0 to 9, with higher scores indicating better development. The instrument has good face validity and good construct validity: factor reproduction of the dimensions, and increasing performance with increasing age (“age trend”). We have used the scales socio-emotional and motor development of WET, because they have been related to the concept of HRQoL and showed a significant relationship with HRQoL in preliminary analysis [29–31]. WET was administered and tested by psychologists and educators using face-to-face interviews with parents and children.

In sum, we collected subjective measures (self-perception), a parental assessment and professional assessments of psychologists and kindergarten teachers to have a more appropriate perspective.

### Statistical analysis

Descriptive analyses were conducted stratifying by gender. Migrant and native-born children were compared with respect to HRQoL as well as the other study variables using t-tests to assess mean differences. We fit a multiple linear regression model with age, gender, positive development and resilience, socio-emotional and motor development, adjusted for socio-economic conditions and area of education. We separated migrants and native-born and investigated the relationship of these factors with HRQoL.

For this study only baseline data were used. Missing data were imputed by substituting for the missing value the scale mean rounded to an integer. Means for all instruments used in the analysis were calculated if up to 33% of responses were missing. For analysis, SAS software was used [32].

## Results

Gender was approximately equally distributed in the present sample (50.9% girls) (Table 1). The mean age in years was 4.25 (SD 0.88) for boys and 4.25 (SD 0.82) for girls. In 72% of families both parents were born outside of Germany, 9.3% had one parent born outside Germany and 18.5% had both parents born in Germany. We did not have the information of origin of 4 families that were part of the study, and who could not be classified.

**Table 1:** **Descriptive statistics of the study sample**

	Boys	Girls
	N = 139	N = 144
	Mean [percentage]	Mean [percentage]
Migrant families	[79.7%]	[83.0%]
Native-born families	[20.3%]	[17.0%]
Age	4.25 (SD 0.88)	4.25 (SD 0.82)
*Area of education*		
St Gallus	[25.2%]	[30.6%]
St Pius	[19.4%]	[14.6%]
St Fidelis	[17.3%]	[16.0%]
St. Martin	[15.1%]	[17.4%]
St. Michael	[23.0%]	[21.4%]
*Origin of migrant families:*		
Asia	[40.2%]	[45.5%]
Africa	[40.2%]	[30.3%]
Eastern Europe	[15.2%]	[5.1%]
Western Europe	[3.3%]	[15.2%]
North and South America	[1.1%]	[4%]

Fathers were predominantly the main economic providers of the family. The origin of the migrant families was heterogeneous. The largest group of children came from Asia (especially from Turkey) with 42.8% and the second largest group from Africa representing 35.2% (mostly from Ghana and Morocco); the third group was from Eastern Europe (mostly from Bosnia, Bulgaria, Russia and Albania) constituting 10.1% of the whole study population (Table 1).

We found statistically significant differences between migrant and native-born children in their socio-economic status (p = 0.00). Results showed migrants to have lower socio-economic levels. But no statistically significant differences were found among groups in the other study variables (Table 2). HRQoL was slightly higher in migrants, but these differences were not statistically significant comparing migrants to native-born. Girls reported higher HRQoL compared to boys, especially in native-born (Table 3). Gender differences were statistically significant on the t-tests in the overall sample.

**Table 2:** **Mean score and standard deviation (SD) of study variables for migrant and native-born children**

Variables	Migrant (N = 227)		Native-born (N = 52)		
	Mean [%]	(SD)	Mean [%]	(SD)	Significance
HRQoL	69.85	(17.00)	68.33	(17.31)	0.39
Positive development and resilience	21.06	(4.15)	21.37	(4.60)	0.52
Socio-emotional development	4.48	(2.58)	4.84	(2.04)	0.54
Motor development	5.89	(2.39)	5.44	(2.64)	0.13
*Socio-economic status*					
Professionals with advanced qualifications	[10.5%]		[40.5%]		0.00
Skilled non manual workers	[11.5%]		[23.8%]		
Skilled manual workers	[47.4%]		[23.8%]		
Unskilled workers	[20.1%]		[4.8%]		
Out of work	[10.5%]		[7.1%]		

Significance measured with t-tests.

**Table 3:** **Mean score and standard deviation (SD) of HRQoL (Kiddy-KINDL) for migrants and native-born and stratifying by gender**

	Total		Boys		Girls	
	N = 283		N = 139		N = 144	
	Mean	(SD)	Mean	(SD)	Mean	(SD)
Migrants	69.85	(17.00)	68.12	(17.64)	71.51	(16.23)
Native-born	68.33	(17.31)	64.29	(17.74)	73.08	(15.87)
Overall	69.09*	(17.51)	66.20	(17.69)	72.29	(16.05)

Note: *Overall was calculated with N = 279 due to missing data at the classification of migrants and native-born; the *t*-test showed statistically significant differences between boys and girls in the overall sample.

Table 4 presents the results of the multiple linear regression. Among migrants, age showed almost significant associations with HRQoL (p = 0.07). By contrast, no differences were observed among migrants and native-born.

**Table 4:** **Multiple linear regression model of HRQoL (Kiddy-KINDL) for migrants and native-born**

	Migrants			Native-born		
	B	SE	t significance	B	SE	t significance
Age	0.47	2.20	0.82	3.73	4.51	0.41
Gender	4.50	2.54	0.07	6.05	5.30	0.26
Positive development and resilience	-0.25	0.30	0.40	0.15	0.58	0.79
Socio-emotional development	-0.11	0.59	0.84	-2.14	1.59	0.18
Motor development	1.08	0.77	0.16	0.32	1.34	0.33

Note: The model was adjusted for area of education and socio-economic status.

## Discussion

The purpose of this study was to assess HRQoL in children predominantly with migrant backgrounds. We hypothesized that despite the possible changes in the migration regime in recent years, migrant children would report lower HRQoL because of the stress of dislocation of the family and their social background. However, we found no important differences in HRQoL scores among migrants and native-born children. If anything, migrant children seemed to report slightly higher perceived health than native-born children, and girls tended to report higher HRQoL compared to boys, especially in native-born children. Our findings differ from those reported in previous studies in which migrants have been found to report worse subjective health compared to the native-born [3, 5]. However, the differences between the instruments applied in the studies limit the comparison of the findings.

We found significant differences in socio-economic status between migrants and native-born children, where migrants had lower levels. Despite these differences, findings are not in line with the results of Pantzer et al. and migrants did not seem to be adversely affected [22]. We found HRQoL levels were not lower in migrant children compared to native-born children. And there were no important differences in the other variables under study between the two groups. These results seem to be different compared to previous other European studies [33, 34], but similar to studies based in the United States [35]. We suggest that the force of selective migration may have changed in recent years. Germany has been receiving skilled workers, who despite their lower socio-economic levels (compared to the native-born) report a high level of perceived health compared with previous migrants [12]. Indeed, selective migration may have become more accentuated following the 2008 economic crisis. However, this hypothesis cannot be tested with the data of our study, since data concerning duration of residence of migrants in Germany was not available. An alternative explanation of the results is based on life course trajectories of HRQoL. Our sample is based on young children and they are normally less affected by differences with peers, do not yet suffer bullying based on their migrant status, and have fewer problems adapting to a new language than older children [36, 37].

Comparing the HRQoL mean score with other studies we see that our scores are lower compared with the scores reported by the German National Health Interview and Examination Survey for children and Adolescents (KIGGS) [20]. The children in our study were approximately 0.5 - 1 standard deviations below the children with migrant backgrounds in the KIGGS study. Anyway, caution is warranted in interpreting these differences because the studies used different versions of the KK, ours based on self-reports, sampled children of different ages, and our study was not based on a nationwide random sample. However, our result is worrisome since studies show a trend towards a decrease in HRQoL with increasing age [38].

Some limitations of the study deserve comment. Our results are based on a kindergarten-based convenience sample from a central highly populated region in Germany. However, we consider our results have internal validity for comparisons between migrant children and native-born German children, i.e. similar selection factors for enrollment in the same kindergartens. The study has several strengths. To our knowledge, this is the first study that investigated HRQoL in kindergarten children predominantly with migrant backgrounds and in a self-reported fashion. HRQoL data was collected from multimodal sources of information: the children themselves, in addition to other data from the parents and the kindergarten teachers.

## Conclusion

To our knowledge, this is the first study that measures HRQoL in young kindergarten children using self-reports that compare migrants and native-born children. Our results suggest that HRQoL at early ages has a different pattern than previous studies among migrant school children. However, these results should be further tested since the duration of residence of families in Germany was not available, and the sample has internal validity, but not external validity. Our findings underscore the need to study the life course trajectory of HRQoL among migrants. Since our study has exploratory character, further replication is warranted in representative samples, not least to improve management of HRQoL in young children with migrant background.

## Abbreviations

HRQL: Health related quality of life
EU: European Union
KK: Kiddy-KINDL
PERIK: Positive Development and Resilience in Daily Life of Kindergarten Children
WET: Vienna Developmental Test
KIGGS: German National Health Interview and Examination Survey for children and Adolescents.
